# Evaluation of the ionization of noble gases under the effect of an electric field for the generation of useful energy

**DOI:** 10.1016/j.heliyon.2024.e25967

**Published:** 2024-02-12

**Authors:** Victor Bonfim Gomes, Robson do Carmelo Santos Barreiros, Pedro Dreyer, Jéssyca Maria Pascoa, Flávio Oliveira

**Affiliations:** IATI - Instituto Avançado de Tecnologia e Inovação, Street Potira, 31 - Prado, Recife, 50751-310, Pernambuco, Brazil

**Keywords:** Wireless power transfer, Ionization, Noble gases

## Abstract

This work originated from the demand presented by an electric power transmission company and addresses a possible solution for the sector by exploring alternatives to extend the flight time of drones in the inspection of transmission lines. This original article demonstrates the use of the electromagnetic field of a transmission line to generate useful electrical power at the terminals of a bulb containing argon gas. It is an unprecedented application in power transmission. In this work, the tests based on a proof of concept are documented, where the results obtained were satisfactory and still allowed to connect an LED through the constructed arrangement. It is observed that the continuity of this research can provide scalability for applications whose main source of ion excitation is given from the energy dissipated as electric field loss in high-voltage lines.

## 1 Introduction

Seeking to meet the demand of a company in the Brazilian electricity sector, which wants to feed quadcopters used to carry out the inspection of transmission lines, we sought to develop a system capable of using the transmission line electric field to generate useful power and thus allow the batteries charging during flight.

The proposed system consisted of a glass bulb, with electrodes at the inner ends, filled with a gas or a mixture of noble gases at low pressure. When the bulb was subjected to an intense electrical field, it's gas was ionized and generated charge carriers.

Due to the charge oscillations inside the bulb caused by the external electric field oscillations, it is assumed that there is a difference in electric potential at its terminals, causing the bulb to function as a current source.

The system proposed in this article stands out for its novelty and originality, so our team has not found a parallel in the literature on the generation of useful electrical energy, which in this case has lit an LED through confined gas usage that has been ionized through an oscillating electric field component without a physical connection between the high-voltage source and the gas.

## 2 Theoretical basis

### 2.1 Transmission lines

**Figure 1:**
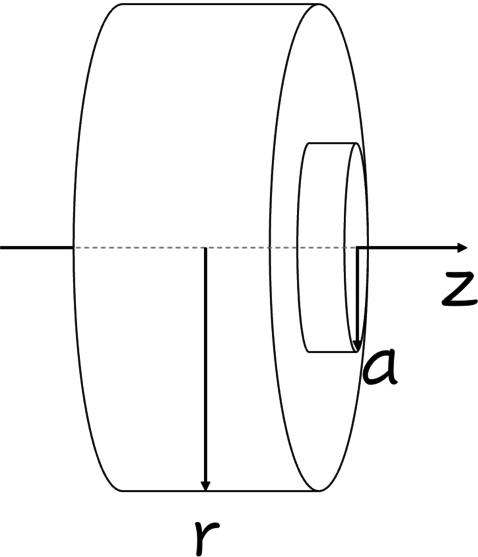
Transmission line cable used in theoretical development.

From the Laplace equation in cylindrical coordinates, we have $\nabla^{2}V=0$ where the boundary conditions are $V\left(a\right)=V_{0}$ e V(b)=0, b>a, for a region outside a cylinder, Fig. 1.

Approaching this cable to a transmission line, which is composed of an infinitely long cylindrical conductor of radius *a*, excited by a source of angular frequency *ω*, we can obtain expressions for the electric and magnetic fields near the line using the classical theory of electromagnetism.

As the propagation mode in the transmission line is electromagnetic transverse, we have that the components in the *z* direction are zero, Equation (1):

(1) $$\nabla^{2}V\left(r\right)=\frac{1}{r}\frac{\partial }{\partial r}\left(r\frac{\partial V}{\partial r}\right)+\frac{1}{r^{2}}\frac{\partial^{2}V}{\partial \phi^{2}}=0$$

As there is an axial symmetry, the term $\frac{\partial^{2}V}{\partial \phi^{2}}$ is zero and the equation reduces to Equation (2):

(2) $$\frac{\partial }{\partial r}\left(r\frac{\partial V}{\partial r}\right)=0$$

The general solution of this equation is given by V(r)=Aln(r)+B, where A and B are constants. Applying the boundary conditions, the expression for the potential is given by the Equation (3):

(3) V(r)=V0ln(ba)ln(br)

The electric field is obtained by deriving the potential as a function of radius, Equation (4)

(4) $$\overset{\to }{E}=-\overset{\to }{\nabla }V\left(r\right)=-{\overset{ˆ}{a}}_{r}\frac{dV(r)}{dr}$$

In which ${\overset{ˆ}{a}}_{r}$ is the unit vector in the radial direction in cylindrical coordinates. The field is given by the Equation (5):

(5) E→=V0rln(ba)e−iβzaˆr

Switching to the time regime, the electric field is given by the Equation (6):

(6) E→=V0rln(ba)cos⁡(ωt−βz)aˆr

### 2.2 High voltage source

**Figure 2:**
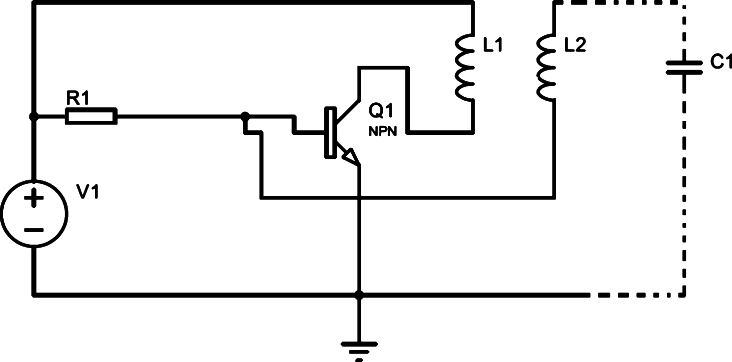
Slayer Exciter Circuit.

In order to reproduce fields and voltages of the same magnitude order as the transmission lines, we use a simple oscillator circuit known as *Slayer Exciter Circuit*, Fig. 2. This oscillator circuit consists of a resistor, a transistor, and an autotransformer. In this circuit, the transistor is responsible for switching as the polarity on the secondary is reversed.

To calculate the electric field near the secondary coil, we need to know the magnetic field flux when a current i(t) flows through the primary. However, as the mutual inductances are equal [1], we can calculate the magnetic flux by passing this same current i(t) through the secondary and calculating the flux in the primary, reaching the same result.

**Figure 3:**
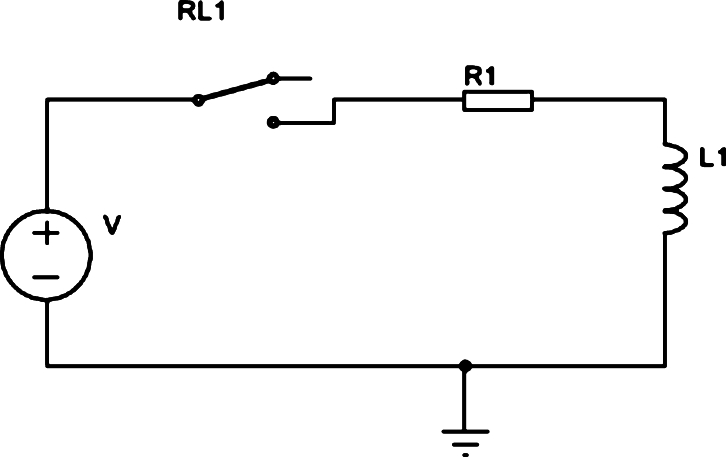
Equivalent circuit for calculating current.

In the circuit of Fig. 2, we have initially that the current and voltage in the coil are zero. This way, we can calculate the current in the primary, i(t), using the equivalent circuit in Fig. 3.

The calculation of the current in the primary from the equivalent circuit in Fig. 3 gives us the Expression (7):

(7) $$i(t)=\frac{V1}{R1}(1-e^{-\frac{R1t}{L1}})$$

In this expression, one can observe the variables R and L, which are the resistance and inductance of the primary, respectively.

Once the expression for current is obtained and considering the secondary coil is very long with very close turns, we can determine the magnetic field inside it using Ampere's law:

$$\oint \overset{\to }{B}\cdot \;d\overset{\to }{l}=\mu_{0}I_{enc}$$

Here we see that $I_{enc}$ is the entire current enclosed by the amperian. From this expression, we get that the magnetic field inside the coil is:

(8) $$\overset{\to }{B}=i(t)n_{2}\mu_{0}{\overset{ˆ}{a}}_{z}$$

So that $n_{2}$ is the number of turns per unit length in the secondary and $\mu_{0}$ is the magnetic permeability of the vacuum.

As a result, the magnetic flux, $\Phi =\int \overset{\to }{B}\cdot \;d\overset{\to }{a}$, is given by the expression:

$$\Phi =i(t)n_{1}h_{1}n_{2}\mu_{0}\pi a^{2}$$

In this expression, *a* is the radius of the primary loop, and $h_{1}$ is the length of this loop. The variable, $n_{1}$, then represents the number of turns of the primary per unit length. Consequently, the mutual inductance is given by:

(9) $$M=n_{1}h_{1}n_{2}\mu_{0}\pi a^{2}$$

### 2.3 Induced electric fields

As the high-voltage source, Fig. 2, generates a time-varying magnetic field, it is expected to induce an electric field, which can be calculated using Faraday's law $\nabla \times \overset{\to }{E}=-\frac{\partial \overset{\to }{B}}{\partial t}$. Leading to the expression:

$$\oint \overset{\to }{E}\cdot d\overset{\to }{l}=-\frac{\partial }{\partial t}\int \overset{\to }{B}\cdot d\overset{\to }{a}$$

For the secondary coil internal region, the above expression results in

(10) $$E_{\phi }2\pi r=-\frac{\partial }{\partial t}B_{z}\pi r^{2}$$

(11) $$E_{\phi }=-\frac{1}{2}\frac{\partial }{\partial t}B_{z}r$$

(12) $$\overset{\to }{E}=-\frac{1}{2}\frac{\partial }{\partial t}B_{z}r{\overset{ˆ}{a}}_{\phi }$$

The equation (12), developed in most elementary electromagnetism courses, is not the only electric field component produced by the coil [2]. In addition to the azimuthal component, Equations (10) and (11), there is an axial component, $E_{z}$, whose module at the center of the coil can be expressed in terms of the voltage, *V*, over the length of the coil, $h_{2}$ [3].

(13) $$E_{z}=\frac{V}{h_{2}}$$

We can express the Equation (13) using the Equation (8) expressed above and the inductance of the secondary, L, is calculated by the Equation (14):

(14) $$L=\frac{\mu_{0}\pi a^{2}N_{2}^{2}}{h_{2}}$$

So we can express the equation (13) as described below in Equation (15):

(15) $$E_{z}=\frac{\pi a^{2}N_{2}}{h_{2}}\frac{\partial B_{z}}{\partial t}$$

An axial component of the electric field near the coil is much higher than the azimuthal component, this must be a charge distribution [3]. In addition, this load distribution generates a capacitive effect responsible for the oscillation of the circuit in Fig. 2, i.e., a self-resonance effect.

### 2.4 Ionization of gases

The ionization of a gas can be associated with several phenomena of different natures, such as particle collision and photon absorption [4].

For the application of interest, the ionization caused by the avalanche effect, also called gas multiplication, is sought, in which an electron with energy greater than the ionization energy of the gas collides with a gas molecule, generating an electron-ion pair. The new electron, in turn, can generate a new electron-ion pair, and so on until the energy dissipates. [5].

Thus, considering a free electron at the origin under the effect of an external electric field in the *x* direction, if the magnitude of the field is sufficient to ionize the gas, this electron will form two more electrons through collisions. Each new electron can produce two more electrons by the same process, increasing the number of electrons as the distance from the origin, *x*, increases [6].

Assuming we have *n* initial electrons, when they travel a distance *dx*, they will generate additional *dn* electrons:

(16) $$\frac{dn}{dx}=n(\alpha -\eta )$$

The general solution of the Equation (16) is given by the Equation (17):

(17) $$n=Ae^{(\alpha -\eta )x}$$

where *α* is the number of ionization generated through collisions per unit length, *η* is the number of recombinations per unit length, and *A* is an arbitrary constant [7].

Assuming a transmission line with , the electric field varies from  to , for a distance from the line  a  of the line [8]. It is expected that the gas ionization rate at low pressure is higher than the recombination rate. Due to the charge fluctuations generated in the ionization, a voltage is expected to arise between the bulb electrodes oscillating with the same frequency as the field generated by the line, 60 Hz, as well as higher orders harmonics.

### 2.5 Electric field due to avalanche effect

If we consider that an electron at the tip of the avalanche is confined to a spherical region of radius *r*, we have that the electric field at the tip of the avalanche is given by:

(18) $$E=\frac{\xi e^{(\alpha -\eta )x}}{4\pi \epsilon_{0}r^{2}}$$

In this expression, *ξ* is the charge of the electron. As the avalanche effect is a diffusive process, we can estimate its mean radial distance by the Equation (19):

(19) $$r=\sqrt{\frac{4Dx}{v_{d}}}$$

where *D* is a diffusion constant and $v_{d}$ is the electron drift rate. Therefore, the equation (18) can be rewritten as:

(20) $$E=\frac{\xi e^{(\alpha -\eta )x}}{4\pi \epsilon_{0}}\frac{v_{d}}{4Dx}$$

The equation (20) shows that the electric field created by the charges increases as the avalanche moves until it reaches a level comparable to the applied field [5].

### 2.6 Choice of gases

As it is of interest that the gas uses the energy absorbed from the field to ionize itself in the most efficient way, gases that have non-radiant excited states, that is, with vibrational and rotational absorption bands, should be avoided [9]. Therefore, monatomic gases were chosen to fill the bulb, which guarantees greater safety due to their stability.

To choose the best gas to use in the bulb, the dielectric strength was considered, which is the voltage applied so that the gas is no longer considered an insulator and starts to conduct. Thus, it is expected that the ideal gas must have a low dielectric strength to be used in this application and remain ionized for a longer time [10].

**Figure 4:**
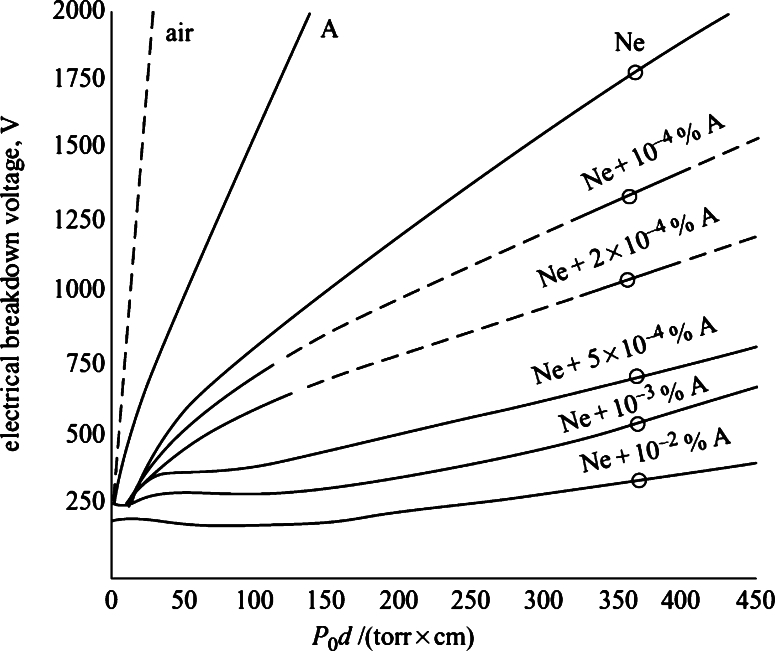
Electrical breakdown voltage, V, in neon-argon mixtures between parallel plates 2 cm apart in 0 ^∘^C, [13].

Thus, we sought to raise gases with lower dielectric strength for a range of 5 to 10 torr⋅cm, in which helium, argon, and neon stand out [11]. However, there is evidence that a mixture of argon and neon gases at different concentrations would present a more effective reduction in dielectric strength, Fig. 4 [5], [12].

The justification for the reduction of dielectric strength lies in the inelastic collision between neon ions and neutral argon atoms, known as Penning ionization. In this process, an atom can only reach its metastable excited state through direct collisions with electrons and, in order to return to the ground state, it must transfer the extra energy to a third body, remaining ionized for a longer time, as in the reaction (21) [14].

(21) $$\mathrm{Ar}+{\mathrm{Ne}}_{m}->{\mathrm{Ar}}^{+}+\mathrm{Ne}+e^{-}$$

Therefore, if the metastable excited state of the atom has energy equal to or greater than the ionization energy of an atom in its vicinity, this process will lead to a system ionization increase and, consequently, to a dielectric strength reduction.

Considering the premises raised above, we propose the hypothesis that it is possible to take advantage of the electric field from a high-voltage line to ionize a gas, resulting in a potential difference that can be reused.

## 3 Experiment and discussion

### 3.1 Construction of the high voltage source

**Figure 5:**
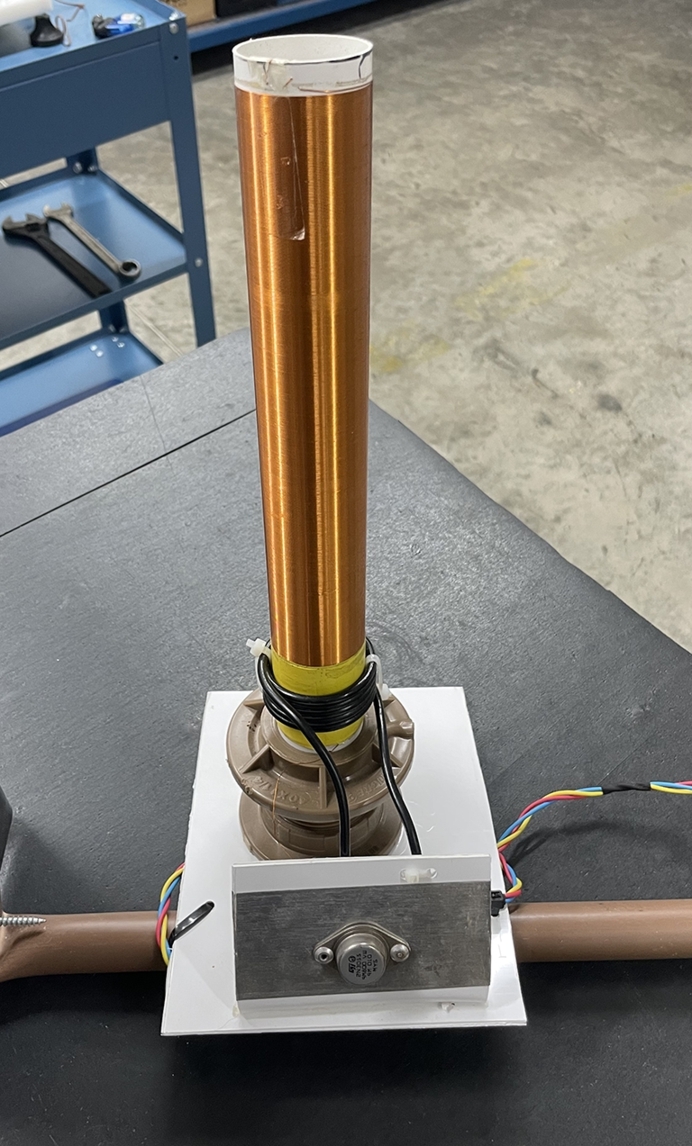
High voltage source manufactured for the tests.

To make the Tesla coil from the *Slayer Exciter Circuit*, Fig. 2, we used 6 AWG and 28 AWG wires for the primary and secondary respectively, Fig. 5. The value of the secondary inductance was calculated using the equation (14).

For the primary inductance, we used the equation (9), and we assumed that all magnetic flux generated by one coil penetrates the other so that the coupling factor is k=1, resulting in the expression (22):

(22) $$L_{1}=\frac{M^{2}}{L_{2}}$$

Height, radius, number of turns, and inductance from the performed calculations are shown in the Table 1.

**Table 1:** Variables from the calculation of mutual inductance.

	Height	Radius	Number of turns	Inductance
Primary	2,5 cm	2,5 cm	6	0,51 *μ*H
Secondary	27 cm	2 cm	844	4,16 mH

The primary terminals were connected to the bench power supply at a voltage of 14,7 , and on the transistor 2N3055 collector. One of the secondary terminals was connected to the base of the transistor, while the other terminal remained open. Finally, a resistor of  was connected between the bench power supply and transistor base, Fig. 2.

### 3.2 High voltage source simulation

Knowing the secondary coil height, $h_{2}$, and radius, *r*, and considering both parameters in meters, we estimated the distributed capacitance in it using Medhurst's formula [15].

$$C=\left(11,26h_{2}+16r+76,4\sqrt{\frac{r^{3}}{h_{2}}}\right)\times 10^{-12}F$$

Therefore, the capacitance distributed across the secondary is C=3,77pF. Once we determine the capacitance, we obtained the circuit oscillation frequency, as shown below.
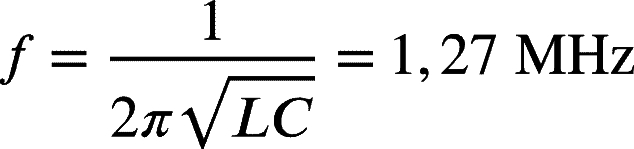


**Figure 6:**
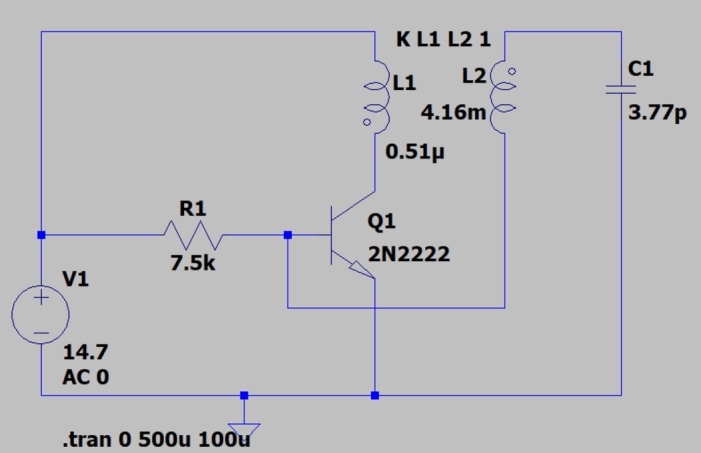
Simulation of the operation of the *Slayer Exciter Circuit*.

Using the LTspice software, we assembled the circuit with the calculated parameters and simulated its behavior, Fig. 6.

**Figure 7:**
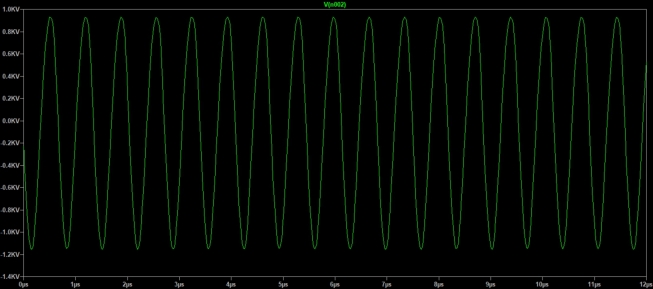
Voltage in the secondary.

**Figure 8:**
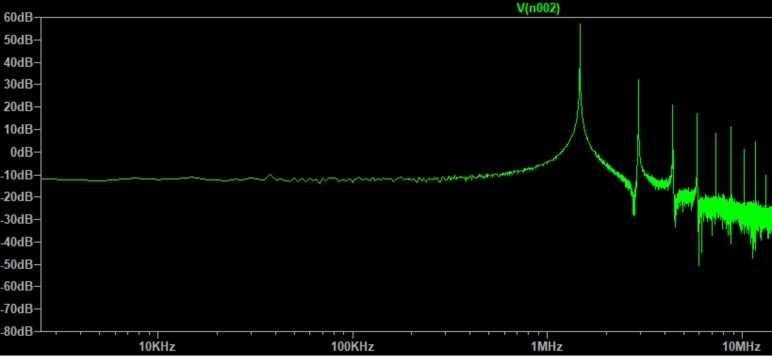
Voltage frequency components in the secondary.

Extracting the FFT, *Fast Fourier Transform*, of the voltage signal on the secondary coil, Fig. 7, we observed that the main frequency component is in 1,46 , Fig. 8. With the expression (12), we estimated the maximum value of the electric field module near the secondary coil using the maximum value of voltage generated in it, Fig. 7, approximately $E_{z}=3,3\;\text{kV/m}$. This value is in the typical operating range of transmission lines.

### 3.3 Bulb manufacturing

In its construction, the bulb for the Electron Flow proof of concept was made with borosilicate glass whose internal diameter is 10 mm with a total length of 170 mm and having the ends enlarged to obtain an internal diameter of 15 mm along 40 mm from each end. In each of the ends, a nickel electrode coated internally with aluminum oxide 30 mm in length and hermetically sealed. These electrodes contain a stainless steel wire terminal each, connecting the electrode to the outside of the bulb.

A capillary tube with 2 mm of internal diameter was fused in the body of the bulb, and this capillary was connected to a system of section valves to remove the internal air by means of a vacuum pump activated for 5 minutes, having the electrode terminals been supplied with an alternating current voltage of 13000 volts per 30 milliamps to eliminate residual air and humidity due to the effect of ionization, before the insertion of the effect gases.

After the preparation process inside the bulb, already free of contaminants and air, the disconnecting switches were switched to introduce the argon gas that was sucked into the bulb by the vacuum previously applied, still remaining inside the bulb at a low negative pressure intensity, having completed the above-mentioned process, the capillary tube was melted for closing and separating the sectional valve system, and it was ready for experimentation.

**Figure 9:**
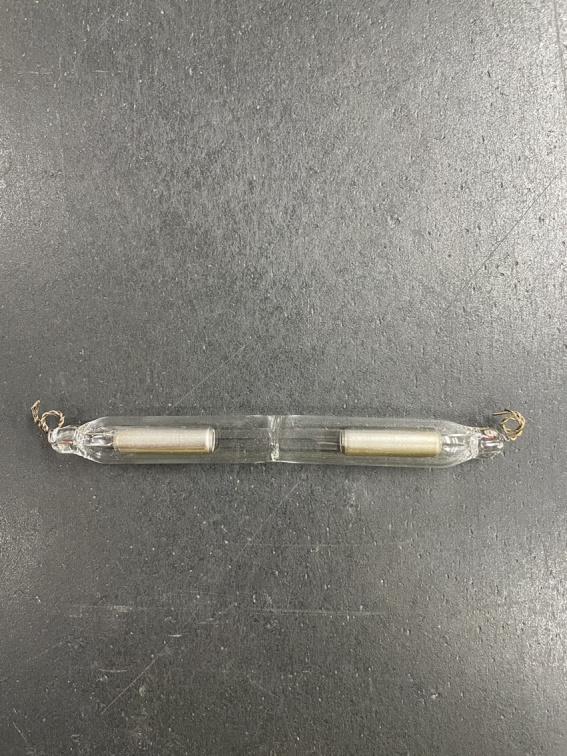
Bulb containing argon at low pressure.

This ampoule was built containing two aluminum terminals, as this metal has one of the lowest work functions among commercial metals [16], in addition to being an inexpensive and highly available material, Fig. 9.

Even though a mixture of neon and argon gases shows evidence of being more efficient, in this proof of concept we used only one gas inside the bulb, argon. The motivation for the use of a gas, instead of a mixture, was given with the objective of promoting a means of guaranteeing the composition of the gases inside the ampoule after its sealing, given the laboratory infrastructure available.

Such a degree of confidence would not be possible if an ampoule were manufactured with any percentage of mixture between argon and neon gas. Other motivations for the use of only one gas in the ampoule are given due to the technical difficulties encountered in the manufacture of the bulb with several gases, which would also require specialized manufacturing infrastructure and which was not an initial part of the scope of this work, being an important point for future developments.

### 3.4 Experimental arrangement

With the values used in the simulation as a base, Fig. 6, we connected the high voltage source that we manufactured, Fig. 5, to a bench power supply with a voltage of 14,7V.

**Figure 10:**
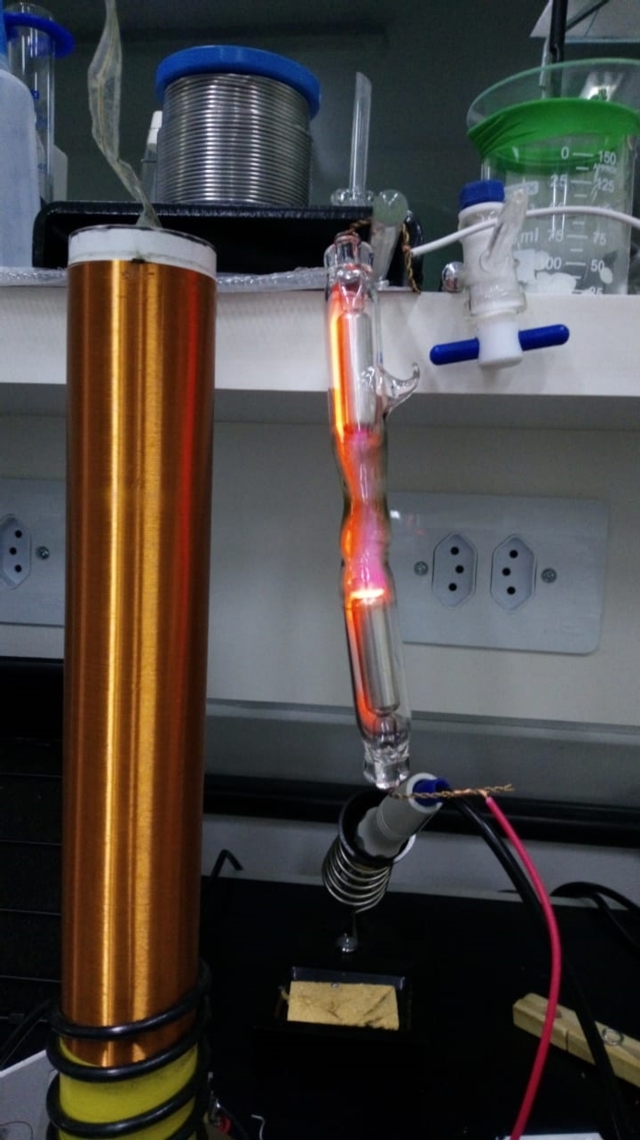
Ionization of gases in the bulb in the presence of an electromagnetic field.

Assuming the principle described in the theoretical foundation that a gas or a mixture of gases at low pressure exposed to an intense electric field ionizes, it was conjectured that when we approach the bulb of the high-voltage source, Fig. 10, a potential difference would arise at its terminals due to the field generated by the charges moving inside it under the influence of the external electric field generated by the source.

**Figure 11:**
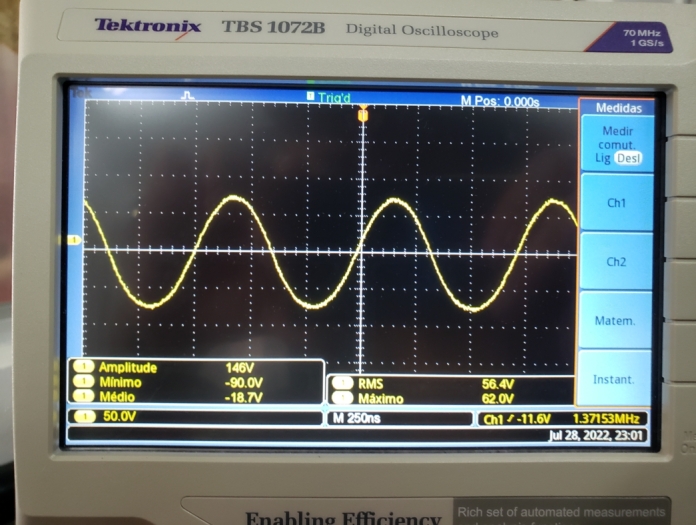
Voltage at the bulb terminals around the high voltage source.

Therefore, the voltage at the bulb is expected to have the same frequency as the high-voltage source. This hypothesis was confirmed by connecting an oscilloscope to the bulb terminals, Fig. 11.

**Figure 12:**
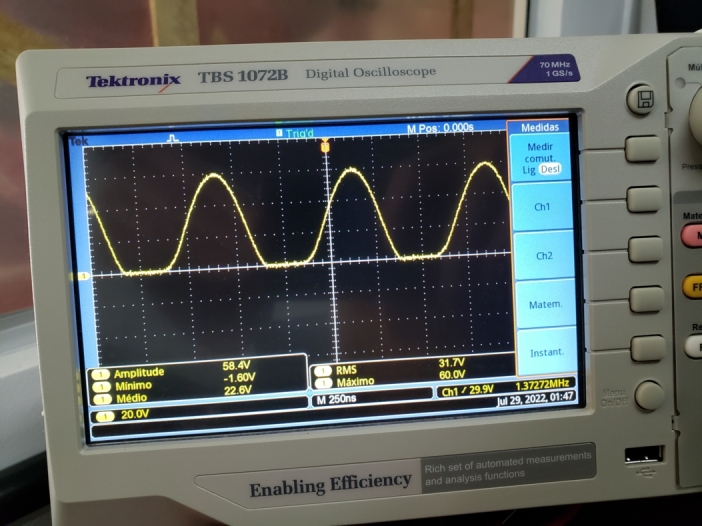
Voltage at the LED terminals connected to the bulb.

When we connected an LED in series with a resistor of  to the bulb, a current of  was observed, and a voltage across the LED terminals, as shown in Fig. 12.

**Figure 13:**
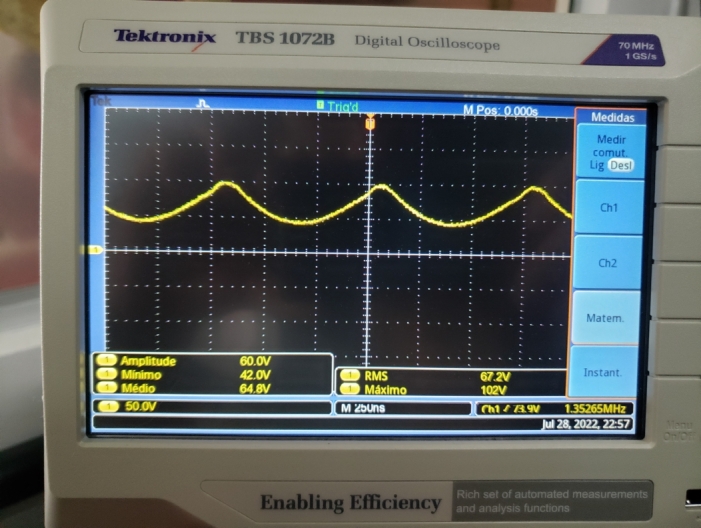
Voltage at the bulb terminals near the high voltage source in series with the LED and resistor.

The voltage across the bulb connected with the LED and resistor can be seen in Fig. 13 in which the frequency of the signal generated in the bulb, 1,35MHz, is the same as the high voltage source and is very close to the values obtained in the theoretical development, 1,27 , and simulated, 1,47 .

**Figure 14:**
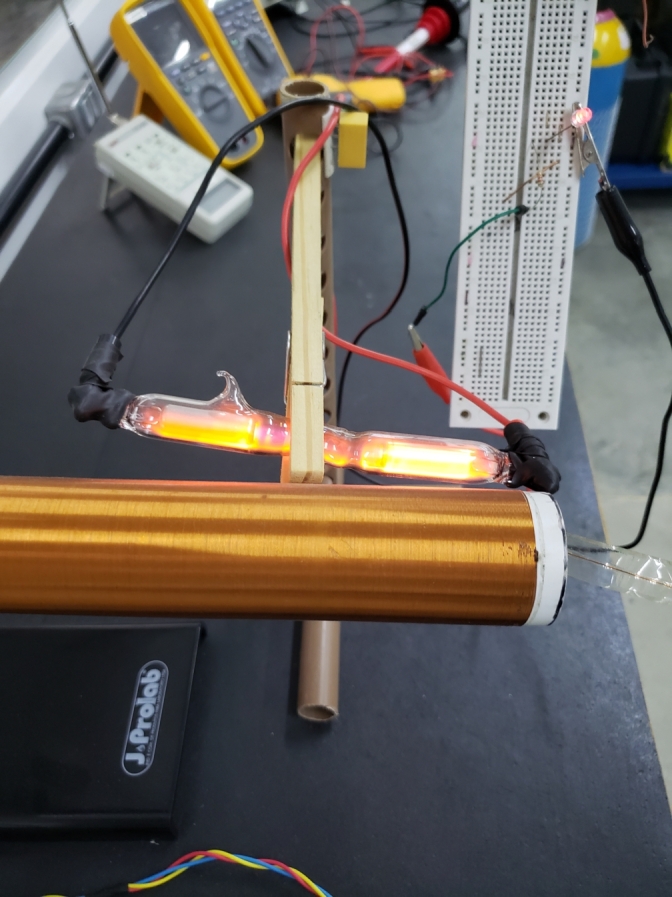
LED on due to ionization in the bulb.

The power consumed by the LED can be estimated from the measured voltage values, Fig. 12, and got close to 0,12μW. Despite being low, the LED lighting confirmed the hypothesis that it is possible to transport energy using ionization mechanisms of gases when these are subject to high-intensity oscillating electric fields, Fig. 14.

## 4 Conclusion

The proof of concept presented in this article validates the thesis that it is possible to use the electric field of a transmission power line through the ionization of a gas, argon, to transmit energy. In the case of this work, we lit an LED. In the future, we intend to study the behavior of other noble gases as well as mixed gases, especially neon and mixtures of neon and argon.

Still, in the future, it is intended to expand the knowledge developed so far in order to increase the performance and spread this understanding to the various applications that are related to energy cogeneration, for example, the capture of energy released as loss in high voltage transmission power lines.

The extent of these studies and research should analyze the best type of modulation of different frequencies of electric fields for the excitation of gases, the best materials that can help in the catalysis of electrons inside the bulb, as well as develop capillarization methodology for accumulator recharge.

## CRediT authorship contribution statement

**Victor Bonfim Gomes:** Conceptualization, Data curation, Formal analysis, Investigation, Methodology, Software, Writing – original draft, Writing – review & editing. **Robson do Carmelo Santos Barreiros:** Conceptualization, Data curation, Formal analysis, Investigation, Methodology, Supervision, Visualization, Writing – original draft, Writing – review & editing. **Pedro Dreyer:** Funding acquisition, Investigation, Methodology, Software, Writing – original draft, Writing – review & editing. **Jéssyca Maria Pascoa:** Project administration, Software, Writing – original draft, Writing – review & editing. **Flávio Oliveira:** Writing – review & editing.

## Declaration of Competing Interest

The authors declare the following financial interests/personal relationships which may be considered as potential competing interests: This contribution is in the context of the project activities of “Inspection with drones by electrostatic coupling for inflight battery charging and use of machine learning for automatic defect classification”, code PD-04825-0006/2019, developed in IATI - Instituto Avançado de Tecnologia e Inovação and funded in the ambit of the Research and Development Program from the National Agency of Electrical Energy (10.13039/501100007133ANEEL) by STN – Sistema de Transmissão do Nordeste.
